# The mental health benefits of visiting canals and rivers: An ecological momentary assessment study

**DOI:** 10.1371/journal.pone.0271306

**Published:** 2022-08-31

**Authors:** Nicol Bergou, Ryan Hammoud, Michael Smythe, Jo Gibbons, Neil Davidson, Stefania Tognin, Graeme Reeves, Jenny Shepherd, Andrea Mechelli

**Affiliations:** 1 Department of Psychosis Studies, Institute of Psychiatry, Psychology & Neuroscience, King’s College London, London, United Kingdom; 2 Nomad Projects, London, United Kingdom; 3 J & L Gibbons, London, United Kingdom; 4 Canal & River Trust, Cheshire, United Kingdom; Forest Research, UNITED KINGDOM

## Abstract

Existing evidence shows positive effects of being in nature on wellbeing, but we know little about the mental health benefits of spending time near canals and rivers specifically. This study investigates the association between visits to canals and rivers and mental wellbeing. We addressed the following questions: Are visits to canals and rivers associated with higher levels of mental wellbeing? Does this association depend on age and gender? Does this association vary between people with and without a diagnosis on mental illness? We used Urban Mind, a flexible smartphone application for examining the impact of different aspects of the built and social environment on mental wellbeing, a strong predictor of mental health. Participants were invited to complete an ecological momentary assessment three times a day for fourteen days. Each assessment included questions about their surrounding environment and mental wellbeing. A total of 7,975 assessments were completed by 299 participants including 87 with a diagnosis of mental illness. Multilevel regression models were used to analyse the data. We found positive associations between visits to canals and rivers and mental wellbeing (p < .05) when compared to being anywhere else and when compared to being in green spaces. Increases in mental wellbeing were still evident after the visit had taken place. These effects remained significant after adjusting for age, gender, ethnicity and education, and were consistent in people with and without a diagnosis of mental illness. Spending time near canals and rivers is associated with better mental wellbeing. These findings have potential implications for mental health as well as urban planning and policy. Visits to canals and rivers could become part of social prescribing schemes, playing a role in preventing mental health difficulties and complementing more traditional interventions.

## Introduction

Spending time in nature is thought to be associated with better mental health outcomes, including better mental wellbeing and lower risk of mental illness [1, 2]. Two types of natural environments have been investigated in the literature: green spaces and blue spaces. The term green spaces refers to areas of trees, plants or other vegetation such as forests, parks and gardens, whereas the term blue spaces refers to all visible bodies of water such as lakes, rivers, canals, ponds, and seas [3]. A growing body of evidence suggests that spending time in nature is associated with mental health benefits, however the existing literature does not typically differentiate between green and blue spaces. Yet there is preliminary evidence that green and blue spaces may have distinct effects on mental wellbeing [4–6] with a recent publication reporting significant effects of green but not blue spaces on mental health [7]. A recent report on possible interventions for anxiety and depression in young people has highlighted the need to investigate the mental health benefits of access to blue spaces specifically [8].

A recent scoping review on the effects of blue spaces on mental health found only five studies investigating the relationship between blue spaces and mental wellbeing [9]. Most of these used a cross-sectional design, involving the acquisition of a single ‘snapshot’, without accounting for the fact that people spend time in different environments throughout the day [10–12]. In addition, most of these studies did not differentiate between the different types of blue spaces and none of them focussed on canals and rivers specifically [10–14].

In England and Wales, people have access to a network of over 2,000 miles (3,218 km) of canals and rivers which connect urban and rural areas. This network is becoming increasingly popular amongst urban dwellers, with 8.3 million visitors in 2020/2021 [15]. Yet at present the mental health benefits of canals and rivers remain speculative in light of its limited evidence base. To overcome this limitation, we adapted a smartphone-based application (app) that has previously been used to explore the effects of the urban environment on wellbeing, to specifically assess the impact of visiting canals and rivers on self-reported mental wellbeing, a strong predictor of mental health in the general population [16]. The app used Ecological Momentary Assessment (EMA), a methodology which involves sampling people’s experiences in real-time and in real-world contexts [17]. This methodology allowed us to explore the relationship between the surrounding environment and mental wellbeing while minimising the risk of recall bias associated with retrospective surveys and questionnaires. The app also collected detailed information on the participants, allowing us to explore how the impact of visiting canals and rivers on mental wellbeing depends on individual characteristics such as age, gender and having a diagnosis of mental illness. A better understanding of the effects of these characteristics is critical for planning and designing urban and rural environments which support mental wellbeing in all citizens. For example, it has been suggested that the mental health benefits of natural environments might differ between individuals with and without a mental health condition [18].

Using smartphone based EMA, we carried out a citizen science study which aimed to answer the following questions:

1. Are visits to canals and rivers associated with higher levels of mental wellbeing? This was assessed by comparing exposure to canals and rivers against a) anywhere else (including all other outdoor and indoor spaces), and b) green spaces. In addition, we considered whether any beneficial effects of canals and rivers on mental wellbeing would persist after a visit has taken place.
2. Does the association between visits to canals and rivers and mental wellbeing depend on age and gender?
3. Does the association between visits to canals and rivers and mental wellbeing vary between people with and without a diagnosis of mental illness?

Additionally, we explored how participants experience visiting canals and rivers by asking them to choose from a list of descriptors (Beautiful, Historic, Ugly, Peaceful, Hectic, Inspiring, Uninspiring, Clean, Dirty, Lively, Dull). These descriptors, selected on the basis that they are commonly used by participants to describe their surrounding environment [18], informed our interpretation of the association between canals and rivers and mental wellbeing.

## Methods

### Design

An observational study using ecological momentary assessments through a smartphone application. The study received ethical approval from the Psychiatry, Nursing and Midwifery Research Ethics Subcommittee at King’s College London (LRS-17/18-6905). Consent was obtained from all participants in written form within the app.

### The Urban Mind app

**Fig 1:**
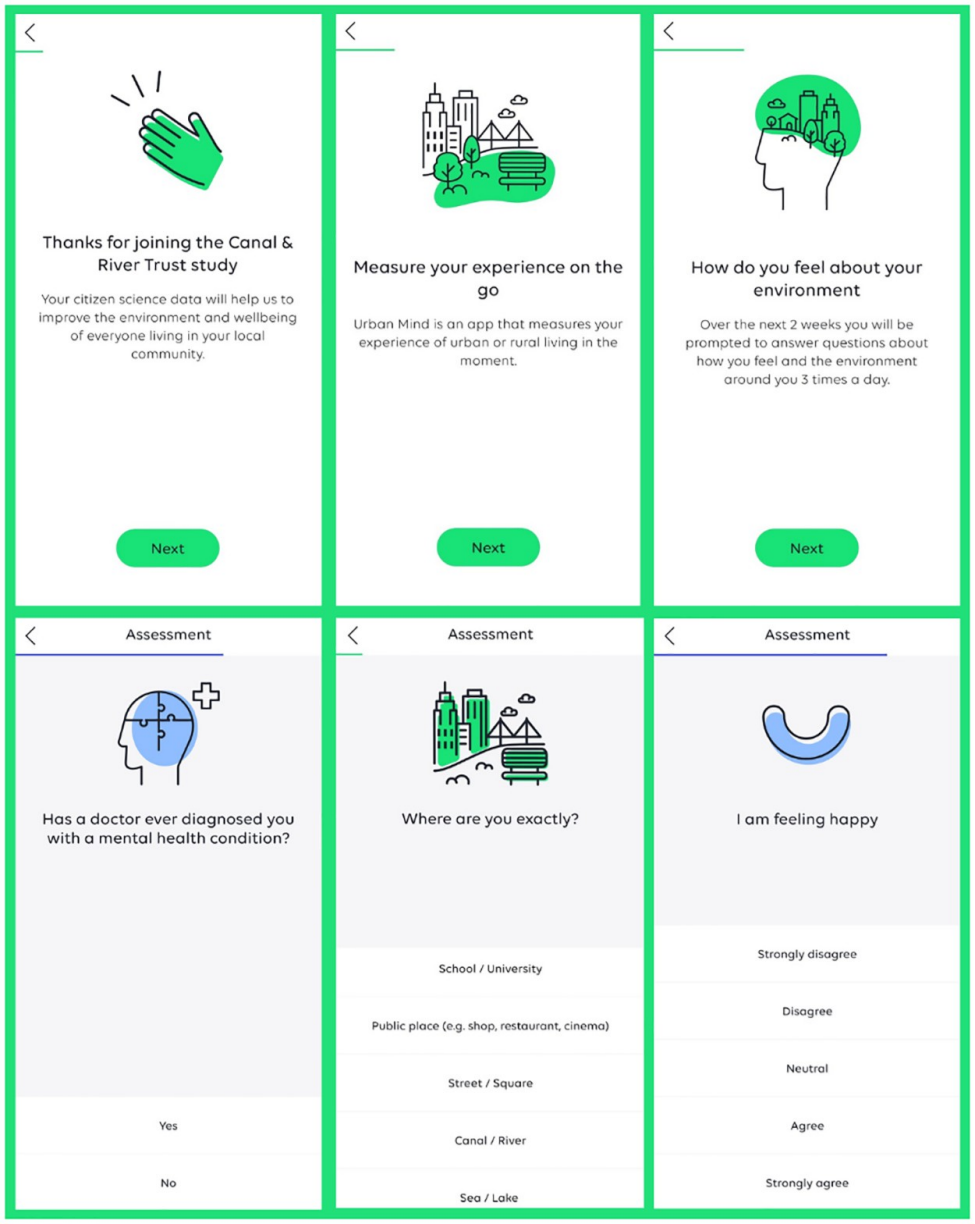
Screenshots of the Urban Mind app interface.

Urban Mind is a smartphone application for measuring the impact of the built and social environment on mental wellbeing, developed by the Urban Mind research project–a collaboration between King’s College London, landscape architects J&L Gibbons and arts foundation Nomad Projects. The app has several versions and has been in use across various projects since 2015 [18]. Urban Mind can be downloaded for free from the App Store and Google Play, and can be used on both iOS (10.3.3 or higher) and Android (7.1.2 or higher) smartphones. Iconography is used throughout the interface to improve engagement and ease of use. Fig 1 illustrates the Urban Mind’s interface.

### Sample

A total of 299 participants were recruited using posters and adverts on the social media and websites of partner organisations (Canal & River Trust, King’s College London, J&L Gibbons, Nomad Projects). In addition, recruitment of participants was promoted with the help of “research ambassadors”. Ambassadors were self-selecting members of the general public who participated in a one-hour online training session introducing the technical and conceptual aims of the study. The training enabled ambassadors to confidently promote participation amongst their local community and to enrol about ten citizen scientist participants each. Their role included liaising between the participants and the core research team, in case any technical or conceptual issues arose while working with the app; and to encourage completion of all the assessments. Ambassadors received an £120.00 honorarium for their time. Individuals were eligible to take part if they were aged 16 and over, spoke English and owned a smartphone with access to the internet. Data collection took place across England & Wales between September 2020 and June 2021.

### Measures

#### Canals and rivers visits

Visits to canals and rivers were measured by asking participants who stated they were currently outdoors to specify the type of place they were in. Participants who selected the option ‘Canal/River’ were coded as 1, with all others, including those that stated they were indoors, coded as 0. Participants were also asked whether they could see water and if so, what type of water they could see (Canal, River, Lake, Pond, Sea, Rain). Those who stated they could see Canal or River were also coded as 1, regardless of the type of place they were in.

#### Past visits to canals and rivers

Participants were asked whether they had visited a canal or a river in the past 24 hours.

#### Green spaces

An additional variable was created to determine visits to green spaces only. Participants were asked to indicate whether they were in a Park, Garden, or Woodland. If participants indicated they could see a canal or a river, they were coded as being near a canal or river, even if they stated they were in one of the defined green spaces.

#### Mental wellbeing

Current mental wellbeing was measured using 10 questions with a 5-point Likert scale (Strongly Disagree, Disagree, Neutral, Agree, Strongly Agree). Five questions referred to positive emotions: I am feeling confident/ relaxed/ happy/ connected to other people/ energetic. Five questions referred to negative emotions: I am feeling anxious/ stressed/ down/ lonely/ tired. Questions referring to negative emotions were reverse-scored and the scores were added up to get a total wellbeing score of 10 (lowest)– 50 (highest). The monitoring of current emotions in order to assess overall mental health is a common and well-established approach in EMA studies, and has been used successfully in several previous studies with clinical and non-clinical populations [19–23].

#### Participant characteristics

Demographic characteristics were measured by asking participants the following questions: ‘How old are you?’, ‘What is your gender?’, ‘What is your ethnicity?’, ‘What is your highest level of education?’, and ‘What is your occupation?’.

The presence of any diagnosed mental illness was established by asking participants to self-report whether a doctor has ever diagnosed them with a mental health condition. Because of small sample sizes, we did not distinguish between the different mental health conditions.

#### Place characteristics

Participants were asked to select how they would describe the place they were currently located. They were asked to select one or more of the following characteristics: Beautiful, Historic, Ugly, Peaceful, Hectic, Inspiring, Uninspiring, Clean, Dirty, Lively, or Dull. In addition, participants were asked to think about the people in the neighbourhood where they were at the time of the assessment and answer the following questions to measure feelings of social inclusivity: ‘Do you feel welcome amongst them?’, ‘Do you feel they would be willing to help you?’, and ‘Do you feel they share the same values as you?’. Participants were considered to feel socially included if they responded ‘Yes’ to at least two of these three questions. Finally, feelings of safety were measured by asking participants whether they would feel safe in the environment they were in a) during the day and b) at night.

## Procedure

Participants downloaded the Urban Mind app on their smartphones and entered the passcode ‘water’ to access the specific research study. Once the app was opened, the participants were presented with several screens explaining the objectives of the study in more detail before being asked to provide informed consent. Participants were then asked to complete a baseline assessment, including questions on their age, gender, ethnicity, education and other sociodemographic characteristics, as well as questions about their physical and mental health. For the next 14 days, participants received push notifications at random intervals three times a day and were asked to complete a short EMA, lasting for around 2 minutes. Once participants received a notification to complete the assessment, they had a 3-hour period of time to do so, otherwise the individual assessment would be marked as incomplete. Participants could access a personalised report any time throughout the study, summarising their experiences as recorded within the app. All data collected by the app were anonymous. Each participant was automatically assigned an ID number which could be used in case they experienced any technical problems. Participants could access the information sheet and privacy policy at any time within the app and had the option to delete all their data and uninstall the app at any time during their participation.

### Statistical analyses

Statistical analyses were performed using STATA 16. Multilevel regression models were used to investigate the association between self-reported wellbeing and being near canals and rivers. First, unadjusted models were calculated, and these were then adjusted for age, gender, ethnicity and level of education. Multilevel models were used to account for having multiple data-points from individual participants. We used a question ‘Have you visited a canal or river in the past 24 hours?’ to investigate whether recent visits were associated with higher wellbeing scores, using regression analyses.

Interaction effects of age and gender were explored by including these variables as interaction terms in the regression analyses. Due to small cell numbers, for the purpose of the statistical analysis, ethnic categories were collapsed into White, Asian (including Asian, Bangladeshi, Chinese, Indian, Pakistani, and Asian other), and Other (including Black African, Black Caribbean, Indigenous other, Arab, mixed, and none of the above). Similarly, having a diagnosed mental health problem was included as an interaction term in the regression analysis.

All models were calculated using data from participants who completed at least 50% of all assessments. Two sensitivity analyses were then ran for each model, including data from participants who completed at least 25% and 75% of the assessments respectively. More information about the models is included in the Technical Note in Supplementary materials (S1 File).

Finally, exploratory χ2 analyses were used to explore how people experience visiting canals and rivers compared to being anywhere else, and adjusted regressions were used to establish the association between place characteristics as described by participants and mental health. All results were considered significant if p < .05.

## Results

The main analysis was based on 6,152 assessments obtained from 186 participants who completed at least 21 assessments each (minimum of 50% response rate). In this sample, 337 assessments were completed near a canal or a river, belonging to 96 participants, whereas 189 assessments were completed while visiting a green space, belonging to 79 participants.

In order to assess the reliability of the findings, we also ran two additional sensitivity analyses including 7,975 assessments obtained from 299 participants who completed at least 11 assessments each (minimum of 25% response rate) and 3,847 assessments obtained from 102 participants who completed at least 33 assessments each (minimum of 75% response rate).

### Participant characteristics

Our total sample of 299 participants included 209 (69.9%) females and 86 (28.8%) males with an average age of 35.97 years (age range: 16–77). The majority of participants (75.9%) identified as white and 72.2% had university education. 144 participants (48.2%) reported they were currently employed or self-employed, 45 (15.1%) were not currently in employment due to retirement or unemployment, and 110 (36.8%) reported their main occupation to be student. The demographic characteristics of participants are presented in Table 1.

**Table 1:** Demographic characteristics of participants.

	>25% completion rate	>50% completion rate	>75% completion rate
	n = 299	n = 186	n = 102
	Number (%)	Number (%)	Number (%)
	Unless otherwise stated	Unless otherwise stated	Unless otherwise stated
Age in years	M = 35.97 (SD = 16.85)	M = 36.26 (SD = 17.29)	M = 36.19 (SD = 17.25)
*Gender*			
Female	209 (69.9%)	126 (67.7%)	69 (67.7%)
Male	86 (28.8%)	57 (30.7%)	32 (31.4%)
Other^a^	4 (1.3%)	3 (1.6%)	1 (1.0%)
*Ethnicity*			
White	224 (75.9%)	142 (77.6%)	83 (82.2%)
Asian	45 (15.3%)	24 (13.1%)	10 (9.9%)
Other^b^	26 (8.8%)	17 (9.3%)	8 (7.9%)
*Education*			
Less than 6^th^ Form	15 (5.0%)	10 (5.4%)	7 (6.9%)
6^th^ Form/Apprenticeship	68 (22.7%)	43 (23.1%)	21 (20.6%)
University	216 (72.2%)	133 (71.5%)	74 (72.6%)
*Employment*			
Employed	144 (48.2%)	89 (47.9%)	47 (46.1%)
Not employed	45 (15.1%)	30 (16.1%)	19 (18.6%)
Student	110 (36.8%)	67 (36.0%)	36 (35.3%)
*Diagnosed with mental illness*			
Yes	87 (29.2%)	57 (30.7%)	36 (35.3%)
No	212 (70.9%)	129 (68.4%)	66 (64.7%)
*Mental health diagnosis* ^c^			
Anxiety disorder	51 (17.1%)	34 (18.3%)	23 (22.6%)
Depression	53 (17.7%)	37 (19.9%)	23 (22.6%)
Bipolar disorder	6 (2.0%)	5 (2.7%)	3 (2.9%)
Psychosis	1 (0.3%)	1 (0.5%)	1 (1.0%)
ADHD	5 (1.7%)	2 (1.1%)	-
PTSD	12 (4.0%)	9 (4.8%)	3 (2.9%)
Other	4 (1.3%)	3 (1.6%)	2 (2.0%)
*Number of comorbid mental health diagnoses*			
1	52 (17.4%)	30 (16.1%)	19 (18.6%)
2	27 (9.0%)	22 (11.8%)	15 (14.7%)
3 or more	8 (2.7%)	5 (2.7%)	2 (2.0%)

Note: numbers and percentages may not add up due to missing values.

^a^ Other (gender): Non-binary and Other.

^b^ Other (ethnicity): Black African, Black Caribbean, Indigenous other, Arab, mixed, and none of the above.

^c^ Categories overlap up as participants may have more than one diagnosis.

### Are visits to canals and rivers associated with higher levels of self-reported mental wellbeing?

**Fig 2:**
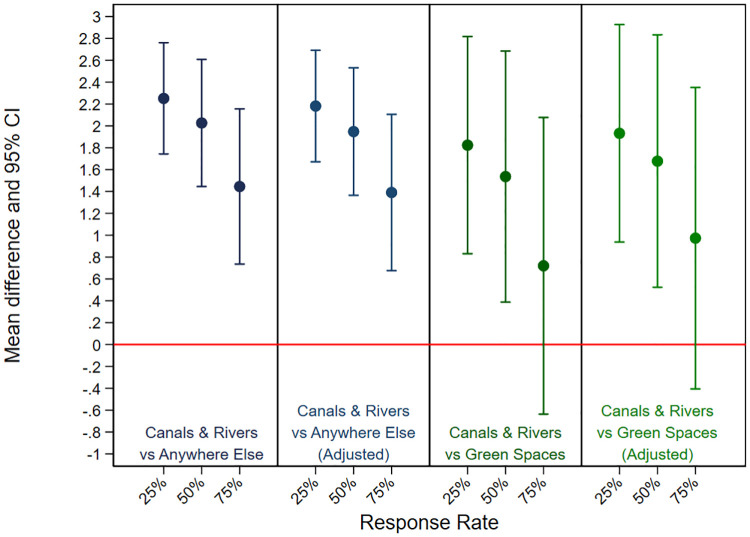
Higher mental wellbeing for visiting canals and rivers compared to being anywhere else and being in other green spaces. Adjusted for age, gender, ethnicity, and level of education. Error bars represent 95% Confidence Intervals.

When compared to being anywhere else, regression analyses showed significant positive associations between visits to canals and rivers and mental wellbeing for each of the three completion thresholds (25%, 50%, 75%). The mean differences (MD–regression coefficients) in the wellbeing scores were 2.25 (95% Confidence Intervals [CI] = 1.74–2.76, p < .05), 2.03 (95% CI = 1.45–2.61, p < .05), and 1.44 (95% CI = 0.74–2.16, p < .05) for the 25%, 50%, and 75% thresholds respectively. The associations remained significant even after adjusting for potential confounders (age, gender, ethnicity, and education). See Table 2 and Fig 2.

**Table 2:** Associations between visiting canals and rivers and subjective mental wellbeing.

Exposure of interest	>25% completion rate		>50% completion rate		>75% completion rate	
	n = 299		n = 186		n = 102	
	Unadjusted	Adjusted	Unadjusted	Adjusted	Unadjusted	Adjusted
	MD	MD	MD	MD	MD	MD
	(95% CI)	(95% CI)	(95% CI)	(95% CI)	(95% CI)	(95% CI)
Being near canal or river vs. anywhere else	2.25*	2.18*	2.03*	1.95*	1.44*	1.39*
	(1.74–2.76)	(1.67–2.69)	(1.45–2.61)	(1.36–2.53)	(0.74–2.16)	(0.68–2.11)
Being near canal or river vs. green spaces	1.82*	1.93*	1.54*	1.68*	0.72	0.92
	(0.83–2.82)	(0.94–2.93)	(0.39–2.68)	(0.52–2.83)	(-0.64–2.10)	(-0.41–2.35)
Visits to canals and rivers in the past 24 hours	1.25*	1.20*	1.35*	1.29*	0.86*	0.82*
	(0.91–1.59)	(0.86–1.55)	(0.96–1.73)	(0.90–1.68)	(0.40–1.32)	(0.36–1.29)
Visits to canals and rivers in the past 24 hours adjusted for current visit	1.24*	1.20*	1.34*	1.29*	0.86*	0.83*
	(0.90–1.59)	(0.85–1.54)	(0.95–1.72)	(0.90–1.68)	(0.40–1.32)	(0.36–1.29)

Note: Mean Difference (MD) and 95% Confidence Intervals (95% CI) represent a mean difference in momentary mental wellbeing score associated with being near a canal or river (exposure) compared to the reference group (being anywhere else, or being in other green spaces) at 25%, 50%, and 75% completion rates. Analyses were explored as crude associations (unadjusted) and adjusted for age, gender, ethnicity, and highest achieved education.

* p < .05.

### How does the effect of canals and rivers on mental wellbeing compare with the effect of green spaces?

Compared to visiting green spaces, visiting canals and rivers was associated with significantly higher levels of wellbeing at 50% threshold with a mean difference of 1.54 (95% CI = 0.39–2.68, p < .05). This difference remained significant after adjusting for potential confounders. These results remained significant at 25% threshold, when the mean differences of was 1.82 (95% CI = 0.83–2.82, p < .05) but not at 75% threshold, when the mean difference was 0.72 (CI = -0.64–2.10). The results are illustrated in Table 2 and Fig 2 below.

### Do the effects on wellbeing persist after the visit has taken place?

Participants who reported they had visited a canal or river in the past 24 hours had significantly higher rates of wellbeing compared to those who did not. The mean unadjusted differences were 1.25 (95% CI = 0.91–1.59, p < .05), 1.35 (95% CI = 0.96–1.73, p < .05), and 0.86 (95% CI = 0.40–1.32, p < .05) for the 25%, 50%, and 75% thresholds respectively. Interestingly, the effects remained significant even when adjusting for current visits to canals and rivers, and when both models were adjusted for confounding variables of age, gender, ethnicity, and education (see Table 2).

### Do gender and age influence the results?

The effect of canals and rivers on wellbeing seems to decrease slightly (MD = −0.06, 95% CI = -0.10 − -0.03, p < .05) with increasing age. The effect was found to be stronger in males when compared to females but only for the 50% (MD = 1.7, 95% CI = 0.45–2.95, p < .05) and 75% (MD = 1.9, 95% CI = 0.36–3.43, p < .05) thresholds. See Table 3 for more details.

**Table 3:** Interaction effects of age, gender, ethnicity, and having a diagnosed mental illness on the association between visiting canals/rivers versus being anywhere else and mental wellbeing.

Interaction term	>25% completion rate	>50% completion rate	>75% completion rate
	n = 299	n = 186	n = 102
	MD	MD	MD
	(95% CI)	(95% CI)	(95% CI)
Visiting canals and rivers * Age	-0.06*	-0.06*	-0.06*
	(-0.08–0.03)	(-0.10–0.03)	(-0.10–0.02)
Visiting canals and rivers * Gender^a^ (Female as reference)	0.70	1.70*	1.90*
	(-0.39–1.18)	(0.45–2.95)	(0.36–3.43)
Visiting canals and rivers *Diagnosed Mental illness	0.14	0.40	1.28
	(-0.92–1.21)	(-0.81–1.62)	(-0.22–2.78)

* p < .05.

^a^ Gender was included as a binary variable (Male/Female).

### Does the association between visits to canals and rivers and mental wellbeing vary between people with and without a diagnosis of mental illness?

We found no significant interaction effect of having a diagnosis of mental illness on the association between visiting canals and rivers and mental wellbeing at any of the three thresholds (50% threshold MD = 0.49, 95% CI = -0.72–1.70). See Table 3 for details.

### How do people experience visiting canals and rivers?

Compared to being anywhere else, participants were more likely to report feeling safe both during the day and at night, as well as feeling socially included when visiting canals and rivers. In addition, they were more likely to describe the surrounding environment as beautiful, historic, peaceful, and inspiring (p < .05). In contrast, participants were less likely to refer to the surrounding environment using negative terms such as ugly, uninspiring, dirty, and dull when visiting canals and rivers (p < .05). No significant differences were found in using the terms clean, lively, and hectic. See Table 4 for details. All results are reported using the 50% completion threshold.

**Table 4:** Description of how people reported experiencing visiting canals and rivers versus being anywhere else and their association with mental wellbeing using the 50% completion threshold.

	Near a canal or river (337 assessments)	Anywhere else (5629 assessments)	Chi^2^	Association with mental wellbeing MD (95% CI)^a^
Safe during the day	98.22%	93.52%	p < .05	0.58 (-0.12–1.28)
Safe at night	83.98%	67.66%	p < .05	0.51* (0.07–0.95)
Socially inclusive	77.74%	70.99%	p < .05	1.45* (1.01–1.89)
Beautiful	58.46%	40.4%	p < .05	1.87* (1.51–2.22)
Historic	28.49%	11.58%	p < .05	0.42 (-0.09–0.92)
Peaceful	65.88%	49.71%	p < .05	0.70* (0.38–1.03)
Inspiring	27.60%	12.61%	p < .05	1.59* (1.11–2.06)
Clean	28.19%	27.59%		0.83* (0.42–1.24)
Lively	10.68%	8.94%		0.53 (-0.12–1.19)
Ugly	1.19%	7.03%	p < .05	-1.54* (-2.21 − -0.87)
Hectic	5.04%	7.44%		-0.78* (-1.35 − -0.22)
Uninspiring	0.89%	9.95%	p < .05	-1.09* (-1.63 − -0.55)
Dirty	0.59%	4.67%	p < .05	-1.93* (-2.74 − -1.12)
Dull	0.89%	8.53%	p < .05	-1.55* (-2.20 − -0.89)

* p < .05.

^a^ Models were adjusted for age, gender, ethnicity, and level of education.

Exploratory regression analyses were conducted to examine the overall association between each place characteristic and mental wellbeing using the whole dataset, with the aim of informing the interpretation of the association between visits to canals and rivers and higher mental wellbeing. This revealed that most positive place characteristics (Beautiful, Peaceful, Inspiring, and Clean) were positively associated with improved mental wellbeing, and that all negative characteristics (Ugly, Hectic, Uninspiring, Dirty, and Dull) were negatively associated with mental wellbeing (p < .05). Similarly, feeling safe in the neighbourhood at night (relative to not feeling safe) and feeling socially included (relative to not feeling socially included) were both positively associated with improved mental wellbeing (p < .05).

## Discussion

Within the existing literature on the mental health benefits of nature, a small fraction of studies has focussed on blue spaces with inconsistent results. While living in proximity of blue spaces has been associated with better mental health [24–26] including lower prevalence of mood and anxiety disorders [27] and schizophrenia [28], several studies have failed to detect significant effects. For example, two recent investigations found a protective role of nature on mental health for green but not blue spaces [7, 29]. Here we expand the existing literature by demonstrating that people experience higher levels of mental wellbeing when visiting canals and rivers compared to being anywhere else. The effect was robust and observed at all three completion rate thresholds as well as after adjusting for sociodemographic variables (age, gender, ethnicity, and level of education). In addition, people who reported having visited a canal or river in the past 24 hours were found to have significantly higher levels of wellbeing than those who did not across the three completion rate thresholds. Interestingly, this effect remained significant even after adjusting for current visits to canals and rivers, suggesting that the wellbeing effect of canals and rivers is long-lasting. While the current study design meant we were unable to establish causality in our findings, the fact that participants reported higher levels of wellbeing not only during but also after their visit to canals and rivers took place supports a potential causal link.

We also found that visits to canals and rivers were associated with significantly higher levels of mental wellbeing than green spaces, but only at the 25% and 50% completion rate thresholds. Because only 102 participants completed at least 75% of the assessments, the lack of significant association found at this threshold may be due to a lack of statistical power. This leads us to the question of why visits to canals and rivers might bear greater mental health benefits than green spaces? A possible explanation is that canals and rivers contain not only bodies of water (i.e. blue spaces) but are almost always associated with an abundance of trees and plants (i.e. green spaces), likely leading to an aggregation of the benefits associated with green and blue spaces [1, 2]. A further explanation might be that canals and rivers provide homes to a range of wildlife, including fish, ducks, herons and other water-dwelling species. Positive association between encountering wildlife and mental wellbeing has been reported by previous studies [30].

The association between visits to canals and rivers was independent of whether the participant had ever been diagnosed with a mental illness. This suggests that individuals with mental health diagnoses, as well as those without, are equally likely to benefit from visiting canals and rivers. In recent years social prescribing of nature-based activities, also known as “green prescribing”, is becoming increasingly popular [31, 32]. Our findings support the view that visits to canals and rivers may be encouraged as part of green prescribing efforts. However, it is important to note that mental health diagnoses were self-reported by participants, who were asked whether a doctor has ever diagnosed them with a mental illness. Future studies should consider using valid and reliable instruments to confirm participants’ current diagnoses and symptoms.

Age and gender both significantly affected the association found between mental wellbeing and visits to canals and rivers, with the strongest effects being found in young people and in males. Young males are at very high risk of developing mental health issues, with as many as one in seven males between the age of 16 and 24 experiencing depression or anxiety [33]. Furthermore, they are least likely to seek help for mental health conditions, resulting in lowest engagement with healthcare services and highest risk of suicide than any other demographic group [34]. Our findings provide a rationale for the future investigations of how prescribing nature-based activities, including visits to rivers and canals, might help address the unmet mental health needs of this underserved group.

When visiting canals and rivers, participants were more likely to use positive place characteristics (Beautiful, Historic, Peaceful, and Inspiring) and less likely to use negative place characteristics (Ugly, Uninspiring, Dirty, and Dull). Participants were also more likely to feel socially included and safe when visiting canals and rivers as compared to being anywhere else. Most positive place characteristics (Beautiful, Historic, Peaceful and Inspiring) were associated with better mental wellbeing, while all negative place characteristics (Ugly, Uninspiring, Hectic, Dirty and Dull) were associated with worse mental wellbeing. Similarly, feeling safe at night and feeling socially included were significantly associated with better mental wellbeing. Taken collectively, these results suggest that the experience of rivers and canals as Beautiful, Historic, Peaceful, Inspiring, Safe and Socially Inclusive may help explain the significant association between canals and rivers and mental wellbeing. Further research is needed to validate and expand these initial findings. In particular, the mediation effects of place characteristics, social inclusion, and perceived safety on the association between visiting canals and rivers and mental wellbeing, could be explored in future studies.

### Strengths

To our knowledge, this is the first study examining specifically the effects of canals and rivers on mental wellbeing using EMA methodology. Previous studies typically grouped all types of blue spaces into one or examined only the effects of being near a sea or ocean [10–14]. However, canals and rivers are more commonly found in urban environments, where access to other blue places may be limited. Furthermore, the majority of previous studies focussed on residential proximity to blue spaces without considering accessibility. Yet there are several instances when people live in proximity of blue spaces with little or no accessibility [35]. Here, using EMA methodology, we were able to monitor actual access to canals and rivers as people went about their daily life. The use of EMA methodology provided dynamic data on participants’ momentary mood and location, allowing us to explore the association between actual visits to canals and rivers and wellbeing in real-time and real-life settings. In particular, we used smartphone-based EMA, which allows for more accurate and complete measurements than traditional methods such as paper diaries and stand-alone devices [36]. While we cannot be certain that the observed differences in wellbeing are due to visits to canals and rivers alone, our analyses were adjusted for known sociodemographic confounders (age, gender, ethnicity, and education) and we also explored interaction effects of gender, age and having a diagnosed mental health condition.

### Limitations

The sample in this study was self-selected, recruited through a limited range of social media and websites. Our sample was likely biased towards people who tend to use the waterways and spend more time in nature. Furthermore, participants were aware of the fact that the study aimed to investigate the impact of being near water on wellbeing, which may have made them more conscious about how they were feeling and therefore their responses may have been biased. However, while the study was advertised as investigating the effects of water on wellbeing, we did also find significant benefits of being specifically near canals and rivers when compared to being in green spaces.

We aimed to overcome some of the recruitment challenges experienced in the previous Urban Mind study [18] by recruiting research ambassadors in order to reach a more diverse population. However, our sample still consisted primarily of white, university educated individuals who were either currently in employment or education. Caution should hence be taken when applying the findings to the general population.

In addition, mental wellbeing was assessed using 10 questions about current emotions using a 5-point Likert scale. While this is a common approach in EMA research, future studies would benefit from using a previously validated questionnaire or validating the questions used in this study.

Lastly, we assessed whether the effects of visiting canals and river on wellbeing were long-lasting by asking participants whether they visited a canal or river in the last 24 hours. A more objective approach would be to conduct lagged analyses, where the association between current mental wellbeing with previous visits to canals and rivers is explored [18]. Due to the relatively small sample size in the current study, and the number of missed assessments per participant, this study was underpowered for this analysis.

## Conclusions

Over half of the world’s population live in cities, a proportion that is expected to rapidly increase in the coming years [37]. Cities are becoming increasingly intensified and engineered with an increasing trend for high-rise buildings and a commensurate decrease in green space [38]. People living in cities may not have easy access to blue spaces such as seas, oceans, lakes, and ponds. Urban waterways (canals and rivers) offer critical access to nature within the urban environment [39]. Our findings suggest that spending time near canals and rivers is associated with better mental wellbeing. Further research on a more diverse sample is needed to allow the generalisation of these findings to the general population and to examine whether visiting canals and rivers causes higher levels of mental wellbeing or whether better mental wellbeing leads to people visiting canals and rivers. If confirmed, the potential impact of the current findings on mental healthcare policy should be considered. Visits to canals and rivers may become part of social prescribing schemes, playing a role in preventing mental health difficulties and complementing more traditional interventions.

## Supporting information

S1 File Technical note. (DOCX)
