# Supplementary material for: The mental health benefits of visiting canals and rivers: An ecological momentary assessment study
Source: PLoS One. 2022 Aug 31;17(8):e0271306. doi: 10.1371/journal.pone.0271306 (PMC9432685; doi:10.1371/journal.pone.0271306)
Supplement: S1 File — (DOCX) [file pone.0271306.s001.docx]

**Technical Note**

**Further details of the statistical analysis**

The study data represents an unbalanced panel, with $N$ (number of individuals) and a variable $T$ (the number of assessments per individual). When exploring the association between mental wellbeing and visits to canals and rivers, let $y_{it}$ be the total score of self-reported mental wellbeing of individual $i$ for assessment $t$. We model these continuous data using a random intercept linear model with…

$$i=1,2,\ldots N$$

$$t=1,2,\ldots T$$

$$y_{it}= \beta_{0}+\beta_{1}{canalsriver}_{it}+\beta_{2}{age}_{i}+\beta_{3}{gender}_{i}+\beta_{4}{ethnic}_{i}+\beta_{5}{educ}_{i}+\varepsilon_{it}+u_{i}$$

…where $y_{it}$ is a linear combination of the following terms. A constant $\beta_{0}$, random intercepts $\varepsilon_{it}$ representing the within-subject error term and $u_{i}$ representing the between-subject error term, ${canalsriver}_{it}$ representing the self-reported exposure to canals or rivers of participant $i$ at assessment $t$, ${age}_{i}$ representing the age of participant $i$, ${gender}_{i}$ representing the gender of participant $i$, ${ethnic}_{i}$ representing the ethnicity of participant $i$, and ${educ}_{i}$ representing the educational status of participant $i$. The parameter of interest, $\beta_{1}$ represents the coefficient of the effect of visiting canals and rivers on total mental wellbeing. Lastly, $\beta_{2}$, $\beta_{3}$, $\beta_{4}$, $\beta_{5}$ are corresponding fixed effects coefficients.

In order to explore interaction effects of age, gender, and self-reported diagnosis of mental illness, we performed similar modelling while including them as separate interaction terms. Let $y_{it}$ be the total score of self-reported mental wellbeing of individual $i$ for assessment $t$…

$$y_{it}= \beta_{0}+\beta_{1}{canalsriver}_{it}+\beta_{2}{age}_{i}+\beta_{3}{gender}_{i}+\beta_{4}{ethnic}_{i}+\beta_{5}{educ}_{i}+\beta_{6}{canalsriver}_{it}*{diagnosis}_{i}+\varepsilon_{it}+u_{i}$$

…where ${canalsrivers}_{it}*{diagnosis}_{i}$ represents an interaction term of self-reported exposure to canals or rivers with self-reported diagnosis of mental illness of participant $i$.
